# Preventing “A Bridge Too Far”: Promoting Earlier Identification of Dislodged Dental Appliances During the Perioperative Period

**DOI:** 10.14740/jocmr1981w

**Published:** 2014-11-19

**Authors:** John T. Denny, Sloane Yeh, Adil Mohiuddin, Julia E. Denny, Christine H. Fratzola

**Affiliations:** a Department of Anesthesia, Rutgers/Robert Wood Johnson Medical School, 125 Paterson Street, New Brunswick, NJ 08901, USA; b Rutgers Graduate School of Nursing, 65 Bergen Street, Newark, NJ 07107, USA

**Keywords:** Partial dentures, Dental injury, Anesthesia complications, Dental complications, Mediastinitis, Cardiac surgery complications, Patient safety, Hand-offs

## Abstract

The presence of fixed partial dentures presents a unique threat to the perioperative safety of patients that require orotracheal intubation or placement of instruments into the gastrointestinal (GI) tract. There are many chances for the displacement of a fixed partial denture: instrumentation of the airway for intubation, or introduction of temporary devices, such as gastroscopes or transesophageal echo probes. If dislodged, the fixed partial dentures can enter the hypopharynx, esophagus or lungs and cause perforations with their sharp tines. Oral or esophageal perforation can lead to potentially fatal mediastinitis. We describe a case of a patient with a fixed partial denture who underwent cardiac surgery with intubation and transesophageal echocardiography (TEE). His partial denture was intact after the procedure. After extubation, he reported that his teeth were missing. Multiple procedures were required to remove his dislodged partial dentures. In sign-out reports, verbal descriptions of the patient’s partial dentures were not adequate in this case. A picture of the patient’s denture and oral pharynx pre-operatively would have provided a more accurate template for the post-operative team to refer to when caring for the patient. This may have avoided the multiple potentially risky procedures the patient had to undergo. We describe a suggested protocol utilizing a pre-operative photo to reduce the incidence of unrecognized partial denture dislodgement in the perioperative period. Because the population is aging, this will become a more frequent issue confronting practitioners. This protocol could mitigate this complication.

## Introduction

The presence of fixed partial dentures presents a unique problem to the perioperative safety of patients that require orotracheal intubation or placement of instruments into the gastrointestinal (GI) tract. There are many opportunities for the displacement of a fixed partial denture. During the perioperative period, when patients have manipulation of their oropharynx to accommodate the placement of an endotracheal tube and/or transesophageal echocardiography (TEE) probe, the fixed partial denture can become dislodged. Likewise, in the post-operative period, if the patient bites or chews on the endotracheal tube, the fixed partial denture can be displaced.

The fixed partial dentures can enter the hypopharynx, esophagus or lungs and cause perforations with their sharp tines. Esophageal perforations can result in mediastinitis with a mortality of 48% [1]. The retrieval process can be traumatic even with endoscopic retrieval: as the denture is pulled out of the GI tract or airway, the tines can rake the mucosal surface, and cause more perforations. In the worst case, the fragile dental appliance can fragment, resulting in multiple smaller, sharp objects which can easily migrate distally. In cases where endoscopy is unsuccessful, thoracotomy is needed to retrieve the fixed partial denture. We introduce and recommend a new safety protocol to reduce the morbidity of a dislodged fixed partial denture.

## Case Report

A 70-year-old man underwent urgent cardiac surgery for coronary artery bypass grafting. Pre-operative assessment showed that his teeth were in poor condition, with the presence of gingivitis. Pre-operative examination of his oral cavity showed a stable fixed partial denture. During the surgery, he had easy, atraumatic orotracheal intubation in one attempt. After intubation, a TEE probe was easily placed in his esophagus and used during the entire case. The TEE probe was moved within the esophagus by routine traction forces applied at the mouth to manipulate the scope - and slide the scope in and out.

The patient remained intubated and sedated at the end of the case and was transported to the cardiac ICU. At that time, the patient had his fixed partial denture in place. A chest X-ray was taken at the time of arrival to the ICU, and there was no evidence of a dislodged denture. During the first 12 h in the ICU, the patient had serial chest X-rays to evaluate his lungs and placement of the endotracheal tube and central lines. He was not unduly agitated. After the patient was extubated, he told the ICU nurses that he was missing his teeth. The patient did not experience dyspnea, coughing or dysphagia. Review of serial chest X-rays confirmed that his fixed partial denture had migrated into his hypopharynx; an unsuccessful attempt was made to retrieve the denture in the hypopharynx and the denture migrated into the esophagus. The gastroenterology service was consulted to remove the denture, but they were unable to retrieve the denture with their endoscope. ENT was subsequently consulted, and they were able to retrieve the denture with a rigid esophagoscope under general anesthesia in the operating room.

## Discussion

The incidence of ingestion of dental appliances after orotracheal intubation is unknown, but it can be compared to information collected on the ingestion of foreign bodies. Some reports state that 1,500 people die annually from the ingestion of foreign bodies [2]. Complications of foreign body ingestion include gut perforation, sepsis, peritonitis, esophagitis, hemorrhage, and impaction of the GI tract. In an unconscious or sedated patient with an unprotected airway, aspiration into the trachea, and commonly the right bronchus, can occur. If not recognized, the fragment(s) can lead to an abscess, and pneumonia. Mediastinitis is already a risk in uncomplicated coronary artery bypass surgery [3, 4]. Perforation in the oral pharynx can lead to cervical necrotizing fasciitis, which can be fatal [5, 6]. As a dislodged device passes distally, esophageal perforation can occur, and also lead to mediastinitis [7]. Esophageal perforation is especially problematic, as mediastinitis has been reported even after botulinum toxin esophageal injection for spasm [8].

Although the term fixed partial denture is used to describe dental appliances, these items can be dislodged if there is no manipulation of the oropharynx. Also, if the patient has poor dentition, it is more likely that the fixed partial dentures can be dislodged from their abutments. The fixed partial dentures contain sharp tines that cause perforations of the GI tract at any point during their migration out of their dental abutments.

A safety protocol to document and emphasize the presence of fixed partial dentures may help reduce the incidence of morbidity related to fixed partial denture dislodgement. In the pre-operative holding area, a digital camera with printer can be made available to document the oral exam of every patient with fixed partial dentures. A picture of the fixed partial dentures *in situ* can be attached to the chart and given as part of patient information during patient verification and sign-out report. The practice of using digital photography to document oral appliances is well known to the dental field; in fact, many employ consumer-level digital cameras over more specialized intraoral cameras as the former are now capable of high resolution capture in macro modes [2]. The old saying of “a picture is worth a thousand words” is apropos here: Being able to show the ICU team a picture of the patients dental appearance pre-operatively is worth more than a verbal description. In our case, despite an adequate description of the patients’ denture to the ICU team, when it subsequently became dislodged, it was only realized when the patient complained. Fixed partial dentures are very hard to verbally describe based on their various shapes, so a verbal description is likely to be inadequate.

The type of procedure should lower the threshold for removing unstable fixed partial dentures. When the patient is undergoing procedures that involve passing instruments repeatedly through the mouth, such as the movement of a TEE during cardiac anesthesia, or the movement of an endoscope for an EGD, the fixed partial denture should be removed if it is slightly unstable. Even if the fixed partial denture is stable, it should be checked periodically during the case for loosening. Furthermore, in cases where the head is not easily accessible to the anesthesiologist, the patient should not be allowed to have the fixed partial denture in his mouth if it is not completely secure. In contrast, if there are no instruments being dynamically placed in the mouth, the clinician can reduce the number of inspections of the fixed partial denture.

In the post-operative period, a detailed report, with pictures, should be given about the location of fixed partial dentures. Furthermore, serial exams of the oropharynx should be done to confirm the location and status of fixed partial dentures. If there is evidence of loosening or displacement of the fixed partial dentures, the post-operative care team should remove the denture or consult dentistry to remove the denture. Special attention should be given to patients who remain intubated postoperatively. In the post-operative care unit, the patient must have adequate sedation so they do not grind their denture on the endotracheal tube and displace their denture. In addition, like the routine monitoring of vital signs, the stability of fixed partial dentures should be verified periodically.

**Figure 1:**
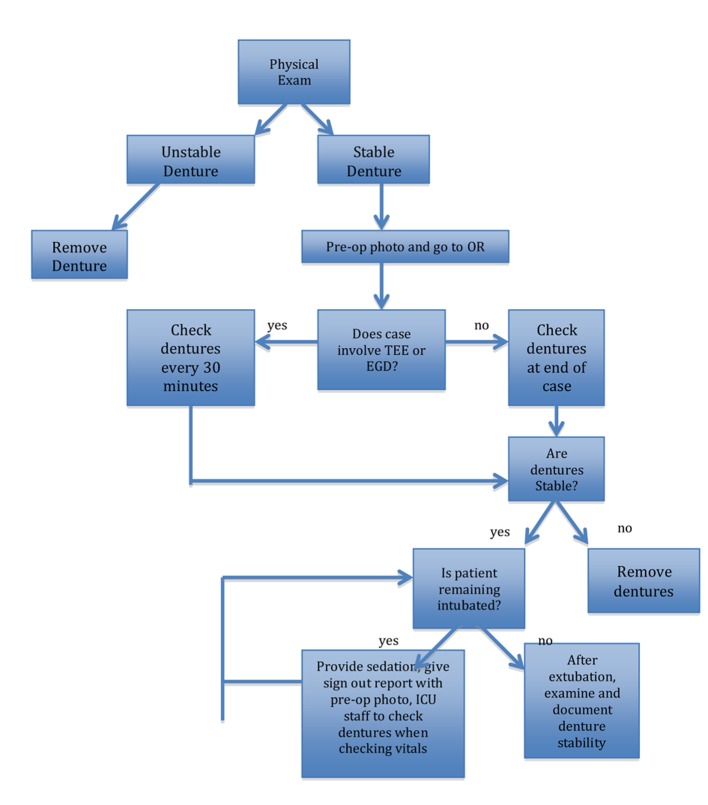
Proposed scheme of management.

Fixed partial dentures have an increasing presence in our aging population. The fixed partial denture has sharp tines which anchor the denture to surrounding teeth. These “fixed” partial dentures have the potential to come loose during general anesthesia when an endotracheal tube is introduced through the mouth to secure the airway. If the patient has a TEE placed in his mouth during a case, it is vital to periodically check on the stability of the denture as a TEE is moved in and out of the patient’s mouth. In addition, when a patient remains intubated at the end of a case, adequate sedation must be provided so that the patient does not bite on his endotracheal tube. With increased surveillance of the fixed partial denture, we can reduce the dislodgement of fixed partial dentures, and avoid the morbidity of the denture traumatizing the GI tract (Fig. 1).
